# Low Shear Stress Upregulates CX3CR1 Expression by Inducing VCAM-1 via the NF-κB Pathway in Vascular Endothelial Cells

**DOI:** 10.1007/s12013-020-00931-4

**Published:** 2020-07-19

**Authors:** Yiwei Zhao, Peile Ren, Qiufang Li, Shafiu Adam Umar, Tan Yang, Yahui Dong, Fengxu Yu, Yongmei Nie

**Affiliations:** 1 Department of Physiology, School of Medicine, Xinjiang Medical University, Urumqi, 830011 Xinjiang PR China; 2 Department of Cardiovascular Surgery, Affiliated Hospital of Southwest Medical University, Luzhou, 646000 Sichuan PR China; 3 Key Laboratory of Cardiovascular and Metabolic Diseases, Affiliated Hospital of Southwest Medical University, Luzhou, 646000 Sichuan PR China

**Keywords:** Human vascular endothelial cells, CX3CR1, Low shear stress, NF-κB, Atherosclerosis

## Abstract

Atherosclerosis is a significant cause of mortality and morbidity. Studies suggest that the chemokine receptor CX3CR1 plays a critical role in atherogenesis. Shear stress is an important mechanical force that affects blood vessel function. In this study, we investigated the effect of shear stress on CX3CR1 expression in vascular endothelial cells (VECs). First, cells were exposed to different shear stress and then CX3CR1 mRNA and protein were measured by quantitative RT-PCR and western blot analysis, respectively. CX3CR1 gene silencing was used to analyze the molecular mechanisms underlying shear stress-mediated effects on CX3CR1 expression. CX3CR1 mRNA and protein expression were significantly increased with 4.14 dyne/cm^2^ of shear stress compared with other tested levels of shear stress. We observed a significant increase in CX3CR1 mRNA levels at 2 h and CX3CR1 protein expression at 4 h. CX3CR1-induced VCAM-1 expression in response to low shear stress by activating NF-κB signaling pathway in VECs. Our findings demonstrate that low shear stress increases CX3CR1 expression, which increases VCAM-1 expression due to elevated NF-κB activation. The current study provides evidence of the correlation between shear stress and atherosclerosis mediated by CX3CR1.

## Introduction

Atherosclerosis, a disease that causes arteriostenosis due to plaque formation, is a leading cause of morbidity and mortality. Although risk factors, such as hypertension and diabetes, lead to vascular endothelial cell (VEC) dysfunction throughout the vasculature [1, 2], atherosclerosis preferentially occurs in arterial regions exposed to low, disturbed, or oscillating blood flow [3]. As a result of the frictional force generated by blood flow, VECs covering the vessel inner surface are constantly subjected to shear stress. Local shear stress alters VEC intracellular signaling, which leads to alterations in gene expression, cell morphology, and structural remodeling [4, 5]. In a healthy human aorta, the average wall shear stress ranges from 10 to 20 dyne/cm^2^ [6]. Normal high arterial shear stress stimulates anti-inflammatory, antiproliferative, and antithrombotic gene expression [7], but low shear stress (±4 dyne/cm^2^) stimulates VECs to release factors that promote cell proliferation, cell adhesion, VEC dysfunction [4, 8], and atherosclerosis [9].

Inflammation plays a primary role in atherogenesis, and numerous studies have shown that the inflammatory response is a key factor in the progression of atherosclerosis in vascular disease [5]. CX3CL1 (Fractalkine, FKN), a CX3C chemokine expressed in endothelial cells, promotes firm cell adhesion and chemotaxis of leukocytes expressing CX3CR1 [10, 11]. While CX3CL1 and CX3CR1 are expressed at low levels in healthy vessels, their expression increases significantly under pathological conditions [12, 13], and pharmacological inhibition of CX3CR1 (the CX3CR1 antagonist F1) has been reported to reduce atherosclerosis [14]. DNA vaccination against CX3CR1 (DEC205-CX3CR1) decreased macrophage recruitment and significant protection from atherosclerosis [15]. In atherosclerotic vessels, the receptor CX3CR1 is expressed on monocytes. Mice deficient in CX3CR1 have previously been employed to investigate the function of monocyte survival and recruitment during atherogenesis [10, 16]. However, we also found CX3CR1 expressed in ECs. Some studies have found the association between CX3CL1–CX3CR1 and atherosclerosis [17]. The CX3CL1–CX3CR1 interaction has been shown to transmit essential survival signals in monocytes and macrophages. The absence of CX3CR1 has been shown to lead to increased death rates, inhibiting atherosclerotic lesion formation [12]. In addition, CX3CR1 plays a role in the formation of atherosclerosis.

NF-κB is a critical regulator of many cell processes, including cytokine production that influences inflammation and stress responses [18]. VCAM-1 is an important adhesion molecule involved in inflammation [19] and its expression on the vascular endothelium can be increased by pro-inflammatory cytokines [20]. Therefore, we investigated the effects of fluid shear stress on CX3CR1 expression in VECs. HUVECs were exposed to different shear stress levels for varying durations to determine mechanisms of low shear stress-induced CX3CR1 upregulation. The results indicate that low shear stress upregulates CX3CR1 expression, which subsequently induces VCAM-1 expression downstream of NF-κB activation in VECs.

## Material and Methods

### Cell Culture

HUVECs derived from EA.hy926 cell lines were purchased from the Shanghai Cell Bank (Shanghai, China) and maintained in HyClone™ Medium 199 (Thermo Fisher Scientific, USA) supplemented with 10% fetal calf serum and 1% penicillin and streptomycin. Cell cultures were grown in a humidified 5% CO_2_ air incubator at 37 °C. Pancreatin (1 mL) was added per culture dish to detach the cells from the plates. Cell suspensions were seeded onto sterile glass slides (75 × 20 mm) coated with 1 mL fibronectin (BD, USA), and were further incubated in empty culture dishes.

### Exposure of EA.hy926 Cells to Shear Stress

A parallel-plate flow chamber system (185 × 95 × 0.8 mm) was incorporated into a closed-loop perfusion system containing serum-free M199 medium driven by a roller pump. Shear stress was calculated according to the equation ${\uptau} = \frac{{6\mu Q}}{{H^2W}}$, here *τ* is shear stress, µ is medium viscosity (0.72 mPa·s), Q is volumetric flow rate, h is chamber height (0.8 mm), and W is chamber width (95 mm). Shear stress of varying levels (0, 2.37, 4.14, 7.11, 9.47, 14.21, and 17.76 dyne/cm^2^) was applied to EA.hy926 cells grown on glass slides for 2 h. The cells were then subjected to a fixed shear stress (4.14 dyne/cm^2^) for different durations (0, 0.5, 1, 2, 4, 6, 8, and 10 h).

### RNA Isolation and qRT-PCR

Cellular RNA was extracted from each individual glass slide using TRIzol Reagent (#15596-026, Life Technology, USA) according to the manufacturer’s protocol. RNA quantity and quality were determined by the A260/A280 ratio using a Nano Drop 1000 spectrophotometer. Two-step reverse transcription (RT) was performed using a RevertAid First Strand cDNA Synthesis Kit (#K1622, Thermo Fisher Scientific, USA) following the manufacturer’s instructions. qRT-PCR was performed using a QuantiFast SYBR Green PCR Kit (cat. no. 204056, QIAGEN, USA). The following primers were used (Shanghai Biological Engineering Company, China): CX3CR1: forward 5’-catcaccgtcatcagcattga-3’, reverse 5’-ggtagtcaccaaggcattcatt -3’; β-actin: forward 5’-ggtagtcaccaaggcattcatt-3’, reverse 5’-ctccttaatgtcacgcacgat-3’. The reaction mix consisted of 2 × QuantiFast SYBR Green PCR Master Mix (10 µL), forward primer (1 µL), reverse primer (1 µL), RNase-free water (6 µL), template cDNA (2 µL), and RNase-free water for a total volume of 20 µL. The CFX96 Real-Time System (BIO-RAD, No. 785BR04347) was employed for this reaction. Relative gene expression was calculated using the 2^−ΔΔCT^ method.

### Western Blot Analysis

Total cellular protein on the glass slides was extracted using RIPA lysis buffer containing protease and phosphorylase inhibitors (Thermo Fisher Scientific, USA). Protein concentration was measured using the Pierce BCA Protein Assay Kit according to the manufacturer’s instructions. Proteins were separated using 12% SDS-PAGE and transferred to polyvinylidene fluoride membranes (LOT 18071300, Roche) by electroblotting. Membranes were incubated in 5% skimmed milk in Tris buffered saline with Tween 20 (TBST) (Boshide, Wuhan, China) for 1 h to block nonspecific binding. Subsequently, the membranes were incubated overnight with either rabbit anti-CX3CR1 (1:1,000; ab8021, Abcam), NF-κB P65 (1:1,000), VCAM-1 (1:2,000), or rabbit anti-GAPDH (1:1,000; Goodhere, China) primary antibodies at 4 °C. The membranes were washed three times with TBST and incubated with secondary antibody conjugated to HRP (1:500; ZSGB-BIO, China) for 2 h. Protein bands were visualized using a color development solution (1:1) for 3 min. Images were acquired using a chemiluminescence imager (Bio-Rad).

### Cell Immunofluorescence Labeling

All incubations were performed at standard laboratory room temperature (22 ± 1 °C) unless otherwise stated. For cell immunofluorescence, the ECs were fixed on glass slides with 4% paraformaldehyde for 1 h and then washed three times using PBS. The cells were blocked with 5% BSA-PBS (Boshide, Wuhan, China) for 30 min, followed by overnight incubation with an appropriate primary rabbit anti-CX3CR1 antibody (1:200 dilution, catalogue no. ab8021, Abcam) at 4 °C. The slides were then washed and incubated for 2 h with secondary biotinylated Alexa Fluor 594 anti-rabbit IgG antibody (1:200 dilution, #8889, Cell Signaling). Studies using these antibodies have been previously published, and related details are available from the manufacturers’ websites.

### Cell Culture and Lentiviral Infection

The culture medium was supplemented with 10% fetal bovine serum (FBS) and 100 units/mL of penicillin and streptomycin. Construct lentiviral vectors and lentivirus were produced by GENECHEM (Shanghai, China). The lentiviruses were constructed encoding sequences targeting CX3CR1 mRNA (siCX3CR1 target-CTTGTCTGATCTGCTGTTT); empty vector was used as the negative control. Stable cell lines were generated by lentivirus infection, which was carried out in a six-well plate with serum-free M199 medium. HUVECs were transduced with lenti-si-CX3CR1 at an infection multiplicity of infection = 10 at 37 °C with 5 µg/mL polybrene and enhanced for 72 h according to the manufacturer’s guidelines. The culture medium was replaced with fresh medium containing 10% FBS, and the cells were continuously cultured for 6–8 days followed by selection with flow cytometry.

### Effect of the NF-κB Inhibitor PDTC on Cell Viability

HUVECs (5.0 × 10^3^/well) were plated and treated in 96-well plates with various concentrations of PDTC (0, 2.5, 5, 10, 20, and 40 µmol/L) for 0, 12, 24, 48, and 72 h. Then, 200 µL of MTT (ZSGB-BIO, China) was added to the cells for 4 h, the MTT fluid was removed, and 150 µL of DMSO (Sigma, USA) was added to the wells. After shaking at a low speed for 10 min, the optical density values of the cells were measured according to the manufacturer’s instructions.

### Statistical Analysis

All results are expressed as mean ± standard error of the mean. Statistical comparisons between two groups were performed using a Student’s *t* test. Statistical comparisons of more than two groups were analyzed using one-way analysis of variance (ANOVA), the least-significant difference test, or multiple ANOVA with SPSS software version 17.0. *P* values < 0.05 were considered significant.

## Results

### Fluid Shear Stress Increases CX3CR1 Expression in VECs

**Fig. 1:**
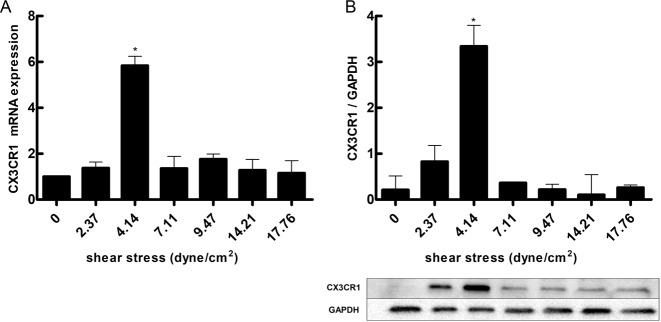
CX3CR1 expression in vascular endothelial cells under different levels of shear stress. **a** mRNA expression of CX3CR1 in vascular endothelial cells subjected to different levels of shear stress. **b** CX3CR1 protein expression detected by western blot analysis in vascular endothelial cells subjected to different levels of shear stress (*P < 0.05 vs. control, *n* = 3 in each group)

**Fig. 2:**
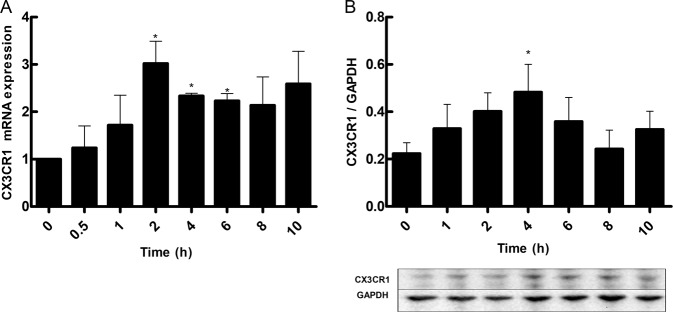
CX3CR1 expression in vascular endothelial cells exposed to different durations of shear stress. **a** CX3CR1 mRNA expression in vascular endothelial cells subjected to different shear stress durations. **b** CX3CR1 expression under different shear stress durations (*P < 0.05 vs. control, *n* = 3 in each group)

**Fig. 3:**
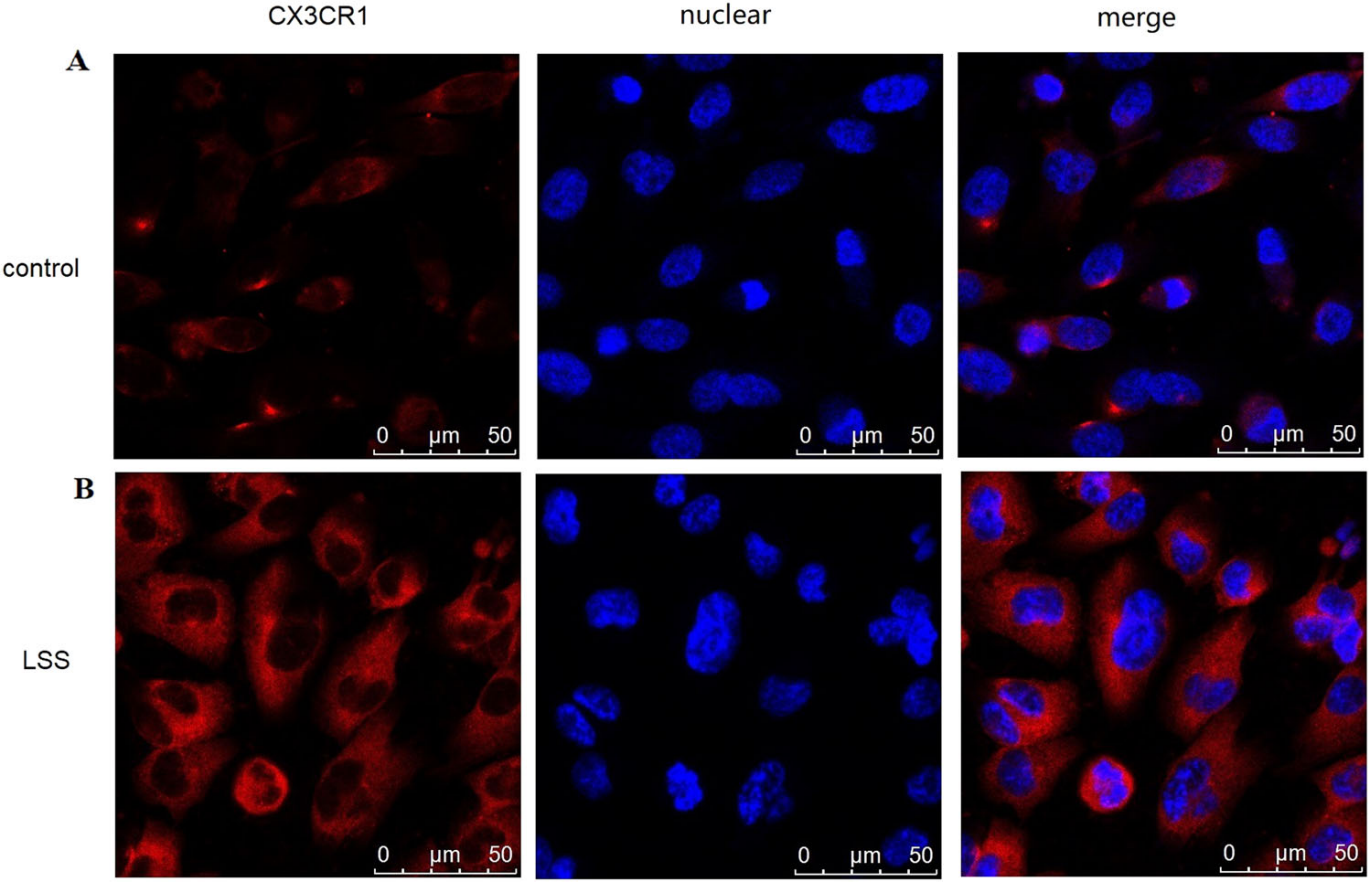
CX3CR1 protein expression remained in the cell membrane after exposure to shear stress. Fluorescence images of CX3CR1 in vascular endothelial cells. The control group (**a**) was maintained under static conditions, and the test group (**b**) was subjected to low shear stress (LSS) at 4.14 dyne/cm^2^ for 4 h

To determine the effect of shear stress on CX3CR1 expression, were exposed to a range of shear stress levels (0–17.76 dyne/cm^2^) for 2 h. We found CX3CR1 expressed in HUVECs. After each experiment, the cells were collected to measure CX3CR1 mRNA levels and protein expression. Both CX3CR1 mRNA levels (Fig. 1a) and protein expression (Fig. 1b) were higher with 4.14 dyne/cm^2^ of shear stress compared with the other shear stress intensities. Then, evaluated the effect of shear stress duration on CX3CR1 expression, cells were exposed to 4.14 dyne/cm^2^ for various durations (0, 1, 2, 4, 6, 8, and 10 h). CX3CR1 mRNA levels in the cells peaked at 2 h (Fig. 2a), and CX3CR1 protein expression in the perfusate peaked at 4 h (Fig. 2b). CX3CR1 protein expression remained in the cell membrane after exposure to shear stress (Fig. 3). These results indicate that CX3CR1 expression is particularly sensitive to low shear stress at 4.14 dyne/cm^2^.

### CX3CR1 Induces VCAM-1 Expression under Low Shear Stress

**Fig. 4:**
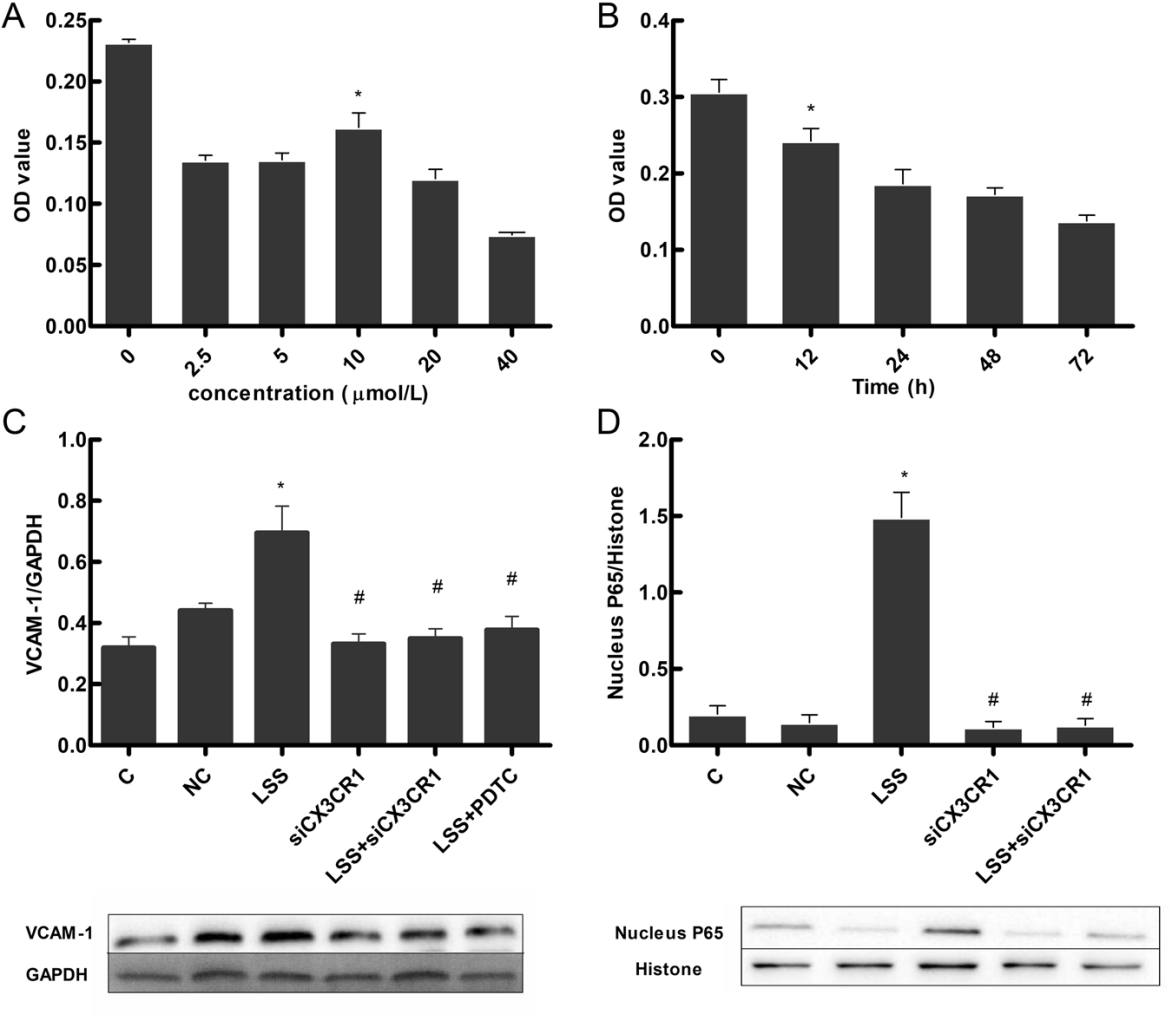
NF-κB mediates CX3CR1-induced VCAM-1 expression under low shear stress. **a** CX3CR1 gene silencing and treatment with the NF-κB inhibitor PDTC reduced the increase in VCAM-1 expression caused by low shear stress. HUVECs were either transfected with specific siRNA against CX3CR1 for 72 h or incubated with PDTC (10 µmol/L) for 12 h; VCAM-1 expression was determined using western blot analysis. **b** PDTC decreased cell viability in HUVECs. Cell viability was assessed using the MTT method. HUVECs were exposed to 10 µmol/L PDTC for 12 h. **c** Inhibition of CX3CR1 reduced the low shear stress-induced increase in P65 expression in HUVECs. **d** HUVECs were transfected with specific siRNA against CX3CR1 for 72 h; nuclear P65 expression was determined using Western blot analysis. (C Control, NC Negative control, LSS low shear stress. *P < 0.05 vs. control; ^#^*P* < 0.05 vs. LSS alone. *n* = 3 in each group)

Studies have indicated that VCAM-1 expression is modified by low shear stress. However, whether VCAM-1 expression is influenced by CX3CR1 under low shear stress has not been reported. Therefore, we determined if VCAM-1 expression was altered in VECs following low shear stress-mediated upregulation of CX3CR1. Western blot analysis showed that VCAM-1 protein expression decreased after transfection with CX3CR1 siRNA, but that VCAM-1 expression increased under low shear stress (Fig. 4a–c). These results indicate that CX3CR1 participates in the vascular inflammatory response induced by low shear stress in VECs.

### NF-κB Mediates CX3CR1-Induced VCAM-1 Expression under Low Shear Stress

NF-κB and its regulatory genes are both directly and indirectly involved in the development of atherosclerosis. Ryan et al. demonstrated that low shear stress can promote nuclear expression of NF-κB [21]. Therefore, we investigated if the NF-κB signaling pathway is involved in low shear stress-mediated upregulation of CX3CR1. We found that downregulation of CX3CR1 by CX3CR1 siRNA decreased expression of the NF-κB protein P65 (Fig. 4d). We further determined that pretreating cells with the NF-κB inhibitor PDTC inhibited CX3CR1-induced VCAM-1 protein expression (Fig. 4c, d). Our data suggest that low shear stress upregulates CX3CR expression by inducing VCAM-1 expression through the NF-κB pathway in VECs.

## Discussion

Mechanical stress is a key factor underlying the pathophysiology of atherosclerosis. When VECs are continuously exposed to blood flow, they are subjected to different degrees of shear stress (resulting from tangential frictional force), including laminar shear stress and disturbed flow, which can affect endothelial cell function [22, 23]. This study provides evidence that low shear stress increased CX3CR1 expression in VECs, which resulted in an increase in VCAM-1 through NF-κB signaling.

Shear stress of 5–12 dyne/cm^2^ is essential to maintain the structural and functional integrity of VECs [24]. Blood flow lower than this range induces pro-inflammatory and pro-thrombotic genes and increases both VEC proliferation and apoptosis [25, 26]. Numerous studies have described the correlation between low shear stress and atherosclerosis. Low shear stress activates the MAPK pro-inflammatory signaling pathway and promotes endothelial cell apoptosis, which is mediated by increased ROS in the mitochondria and cytoplasm and can further activate the serine/threonine kinase Akt in VECs [27]. Cheng et al. found that low shear stress increased IL-8 levels by JNK1/2, ERK1/2, and p38 MAPK [28, 29]. Our results indicate that low shear stress (4.14 dyne/cm^2^) can also increase CX3CR1 expression that is dependent on NF-κB activation in EA.hy926 cells. CX3CR1 belongs to a family of G protein-coupled receptors, at the same time, we also subjected CX3CR1 protein were expression remained in the cell membrane after exposure to shear stress.

Our current findings support our previously published study demonstrating that CX3CL1 expression increases under low shear stress (4.14 dyne/cm^2^) [30]. However, we also found no significant difference about CX3CR1 expression varied with increasing levels of shear stress. Some study found that CX3CL1 expression were downregulated by high laminar shear stress in endothelial, and this were mediated by TNFα [31]. We previously demonstrated that CX3CR1 levels influence atherosclerotic plaque development in rabbits. Furthermore, we reported that shear stress affected FKN expression in VECs, as well as increased ERK1/2, p38, and JNK activation in a time-dependent manner [32]. Inhibiting this activation resulted in downregulation of FKN expression induced by low shear stress. These data demonstrate that low shear stress-induced CX3CR1 expression is closely related to the pathogenesis of atherosclerosis.

Subsequently, we investigated the mechanism by which low shear stress-induced CX3CR1 expression. We found that VCAM-1 increased in response to low shear stress after CX3CR1 silencing. Some findings indicated that atherosclerosis promoted low shear stress by activating the soluble form of VCAM-1 [12]. A previous study found that shear stress plays differential roles in modulating TNF-α-induced VEC expression of VCAM-1 [33]. Therefore, we determined if VCAM-1 expression was downstream of low shear stress-mediated CX3CR1 upregulation. CX3CR1 is a key factor in the vascular inflammatory response induced by low shear stress, and NF-κB is activated upon extracellular stimulation by certain cytokines, inflammatory factors, growth factors, and even specific physical factors [34]. Moreover, we found that low shear stress significantly increased NF-κB P65 expression in the nucleus. siRNA mediated CX3CR1 knockdown decreased NF-κB P65 expression similar to what we observed for VCAM-1 after treatment with the NF-κB antagonist PDTC and low shear stress. Taken together, our study indicates that NF-KB is activated by the CX3CR1 ligand in response to low shear stress.

In conclusion, Our study demonstrates that low shear stress increases CX3CR1 expression and that CX3CR1-induced VCAM-1 expression is mediated by the NF-κB signaling pathway in response to low shear stress. The current data provide evidence of the correlation between shear stress and atherosclerosis that is regulated by CX3CR1.

**Suggested reviewers:** Zuzanna Rowinska: z.rowinska@klinikum-bochum.de. Rachana Shah: shahr@email.chop.edu. Andreas Ludwig: aludwig@ukaachen.de.
